# In search of vitamin sleep: cross-sectional analysis of vitamin and mineral intake and sleep health outcomes in adolescents

**DOI:** 10.3389/fnut.2026.1978612

**Published:** 2026-10-16

**Authors:** Sarah L. H. Kamhout, Sarah G. Bellini, Steven G. Luke, Kate J. Homer, Mary Broadbent, Kara M. Duraccio

**Affiliations:** 1 Department of Psychology, Brigham Young University, Provo, UT, United States; 2 Department of Nutrition, Dietetics, and Food Science, Brigham Young University, Provo, UT, United States

**Keywords:** adolescence, micronutrients, nutrition, sleep health, vitamins & minerals

## Abstract

**Objectives:**

Many U.S. youth fail to meet nutritional guidelines and often struggle with inadequate sleep. We examined how adolescent micronutrient intake is related to sleep. We hypothesized that higher intake of folic acid, potassium, calcium, magnesium, vitamin D, vitamin K, and iron would be associated with earlier chronotype, reduced daytime sleepiness, earlier DLMO timing, and less circadian misalignment. We also explored the relationship between sleep health outcomes and sodium, zinc, copper, selenium, vitamin C, niacin, vitamin B2, vitamin B6, folate, retinol, vitamin B1, vitamin B2, vitamin B12, vitamin A RAE, vitamin E, alpha carotene, beta carotene, lycopene, cryptoxanthin, and Lutein + zeaxanthin intake.

**Methods:**

Across two paradigms, 80 participants (ages 14–18) completed ASA-24 dietary recalls on 2–3 randomized days (weekday and weekend), dim light melatonin onset (DLMO) protocols, sleep health questionnaires, daily sleep diaries, and actigraphy. Using step-wise mixed effects models nested by subject, we explored connections between same-day micronutrient intake and same-night sleep, as well as average relationships across all recall days.

**Results:**

No micronutrient emerged as significantly associated with improved sleep health across all models. Potassium (b's = −0.03 to 0.004, *p's* < 0.05) and retinol (b's = −0.20 to 0.01, *p*'s < 0.05) showed the most consistent associations with positive sleep outcomes. Zinc (b's = −0.54 to 3.51, *p*'s < 0.10), and vitamin B12 (b's = −0.96t to 7.22, *p*'s < 0.05) were most consistently linked with negative sleep outcomes.

**Conclusions:**

These findings highlight potential relationships between micronutrient intake and adolescent sleep health. This can inform further research establishing causality and informing dietary-based sleep health interventions.

## Introduction

According to the National Health and Nutrition Examination Surveys (2015–2016), less than 2% of youth ages 14–18 met recommended vegetable intake guidelines, with 88–99% of children and adolescents consuming less than 0.9 cups of vegetables per day (1). Findings are similar for fruit intake, with 81% of the US population failing to consume the recommended 1.5 cups per day (mean ~0.9 cups; 1). With only 2.9% of children and adolescents meeting daily whole grain requirements (2), and over 90% of the American population over 1 year old exceeding refined carbohydrate limits [(3); page 30], it is possible that both underconsumption of whole foods and overconsumption of processed foods can contribute to micronutrient deficiencies (4). Adolescents may be especially susceptible to micronutrient deficiencies due to their higher consumption of empty calories, which can be driven by taste, social demands, marketing, and income/access (5–7).

Alongside this risk for inadequate micronutrient consumption, it is also well established that adolescents experience reductions in sleep health compared with other developmental groups. Specifically, recent estimates suggest that nearly 70% of adolescents do not obtain the 8–10 h of sleep recommended for their age (8), and over 40% of US adolescents report sleeping less than 7 h per night (9). Short and poor quality sleep in adolescence may be due to the onset of puberty and its associated effects on circadian timing (10), combined with increased independence in setting schedules (11), social pressures such as early school start times (12), and increased screen use (11, 13). It is also possible that deficits in certain micronutrients may contribute to some aspects of adolescent sleep health.

Although much is known about the impact of sleep on dietary behaviors (14), such as poor sleep increasing intake of high-glycemic foods (15), or greater levels of circadian misalignment predicting shifts in appetitive hormone levels (16), less is known about the influence of dietary intake on sleep. To our knowledge, no studies have examined relationships between broad micronutrient intake and sleep outcomes in adolescents. A growing literature base suggests correlational and causal relationships between micronutrient consumption and sleep in adults. For instance, greater folic acid consumption has been linked with higher sleep quality in adult women who are currently pregnant (17), and lower folic acid consumption has been linked with elevated risk of severe difficulty falling asleep in the general adult population (18). Higher levels of potassium, calcium, and magnesium intake have also been associated with higher sleep efficiency and earlier sleep midpoint (19, 20), and lower intake of calcium, magnesium, vitamin D, and vitamin K has been associated with shorter sleep duration (21). Similarly, iron deficiency has been frequently associated with sleep disorders, and iron supplementation is often beneficial in alleviating sleep disorder symptoms [e.g., RLS; (22)]. Other vitamins, such as vitamin B12, demonstrate mixed findings (23–26), suggesting a need for additional clarifying studies.

In addition to vitamin and mineral intake, antioxidant intake is also potentially relevant to sleep health, due to reductions in oxidative stress and inflammation having the potential to drive improved sleep outcomes (27). For example, one study found that adults who had the highest carotenoid intake had lowered risk for poor sleep as compared with those at lower intake levels (28). Additionally, a potential relationship may exist between carotenoid intake and obstructive sleep apnea (OSA), with carotenoid intake being lower on average in patients with OSA (29). An emerging body of research also suggests that carotenoid intake may reduce the negative impacts of blue light exposure on sleep initiation or maintenance (30). Together, these findings can suggest that micronutrients may play a role in current sleep health, as well as future disease prevention.

Although previous research has begun to elucidate how micronutrient intake may be related to sleep outcomes, existing studies have typically been limited to adult samples, single dietary assessments, and single-construct self-report sleep measures. Examining these associations in adolescents is important not only because of the unique risks for poor sleep and diet in this age group, but also due to the potential benefits of early intervention for disease prevention. Examining multiple facets of sleep health can also illuminate ways in which micronutrients may affect adolescents uniquely, as well as ways in which different micronutrients may differentially affect various components of sleep, informing more tailored interventions. Although sleep duration (the elapsed time between sleep onset and wake) is the most frequently studied aspect of sleep health (31, 32), other aspects of sleep such as efficiency (time asleep/time in bed), and time spent wake after sleep onset (WASO) have also been related to micronutrient intake (33, 34). Furthermore, chronotype, which is an individual's innate preference toward early- or late- sleep and wake times, has been related to calcium, magnesium, zinc, vitamin D, riboflavin, and B6 consumption in young women (20). Additional sleep health dimensions including circadian timing, circadian misalignment, and daytime sleepiness may further serve as relevant sleep outcomes.

In this secondary analysis of data from two adolescent studies, we aimed to measure whether micronutrient intake was related to same-night actigraphy-measured sleep onset time, sleep midpoint time, total sleep duration, sleep efficiency, and WASO. To account for day-to-day variation in eating and sleep behaviors (35, 36), knowing that sleep and dietary behaviors on individual days inform broader patterns via autocorrelatory and compensatory mechanisms (37–39), and recognizing that micronutrients vary in terms of intake-dependent bioavailability and long-term storage patterns (40–42), we also explored associations between average micronutrient intake across 2–3 dietary recall days and trends across daily sleep outcomes, as well as with more trait-like facets of sleep health, such as chronotype, average levels of daytime sleepiness, DLMO time, and circadian misalignment. Based on the adult literature, we hypothesized that increased micronutrient consumption in folic acid, potassium, calcium, magnesium, vitamin D and K, and iron would be associated with earlier sleep onset and midpoint times, increased sleep duration and efficiency, and decreased wake after sleep onset during the associated night. We also hypothesized that increased micronutrient consumption in folic acid, potassium, calcium, magnesium, vitamin D and K, and iron averaged across the observation period) would be associated with earlier chronotype, decreased levels of daytime sleepiness, earlier DLMO timing, and decreased levels of circadian misalignment. Finally, as the ASA-24 also collects micronutrient data which is less studied in existing sleep literature but may also influence adolescent sleep health, we also explored how consumption of sodium, zinc, copper, selenium, vitamins A, C, & E, folate, retinol, thiamine, riboflavin, niacin, vitamins B 6, & B12, beta-carotene, beta-cryptoxanthin, and lutein + zeaxanthin relate to nightly and averaged sleep outcomes.

## Methods

All procedures used in this study have been approved by the sponsoring university's Institutional Review Board.

### Participants

Participants were recruited using social media posts, digital flyers included in local high school and university email newsletters, physical flyers posted across the community, and study staff-led recruitment visits to local high school classes and youth groups. Qualtrics surveys were used to determine eligibility for interested participants. Eligibility criteria included current age between 14–18 years and current enrolment in junior or senior high school. Data for this secondary analysis were attained from two separate data pools, from studies conducted between 2022 and 2023, across both academic and summer months. Both samples were recruited from the general population of community adolescents. Given the use of magnetic resonance imaging (MRI) tasks in both studies, those who were not considered MRI-compatible were excluded from the study (e.g., metal implants, pregnancy, left-handedness, and claustrophobia). Finally, those with previous bariatric surgery, eating disorders, severe food allergies, a BMI percentile > 95, and medication that impacted diet were excluded, as outcomes for other analyses in the primary studies pertained to obesity prevention.

### Procedures

At the start of both study protocols, participants completed a baseline visit, where they engaged in consent procedures and received wrist-worn actigraphs (Actiwatch 2). They also completed baseline surveys of chronotype and daytime sleepiness via Qualtrics during this lab visit. Participants were taught how to appropriately wear actigraphs during sleep. Those participating in a protocol requiring all-day wear were asked to don devices at the start of each day. Those participating in a protocol requiring nighttime wear only were instructed to put devices on when entering bed with intention to sleep and take them off when exiting bed in the morning. Study staff instructed participants on how to maintain daily sleep diaries that were used to assist with accurate actigraphy scoring. Participants across the two studies wore actigraph devices at home for 7–20 days. Before leaving the lab, participants were briefed on dietary recall procedures, given access to standard training videos for dietary recall processes, and told to wait for notification to complete dietary recalls on 2–3 (depending on study protocol) randomized days on which they were wearing accelerometer devices. Assigned recall days were randomized to include weekend and weekdays, to account for potential variation in sleep or dietary patterns in response to school or work schedules. Participants were notified of their assigned recall day live (as opposed to in advance) and asked to record dietary consumptions over the past 24-h period (i.e., “please record your food and beverage intake for the preceding 24-h period”) in order to prevent alteration of dietary patterns in response to known observation. Throughout the course of the study, participants received daily text reminders to wear the accelerometers and to complete the sleep diary.

After an at-home sleep stabilization period of 3–5 days, where participants slept according to an unmonitored, self-selected, preferred schedule aligned with their typical sleep behaviors, each subject completed an in-lab six-hour Dim Light Melatonin Onset (DLMO) protocol. The at-home stabilization period was employed to increase the likelihood of obtaining a valid DLMO estimate, as sleep is known to be highly variable within this developmental population (10). Each DLMO appointment occurred on a Friday or a Saturday and began 5 h before and ended 1 h after subjects' self-identified preferred bedtime, to ensure observation of melatonin saturation. Saliva samples were collected every 30–45 min and light levels of < 5 lux were maintained across the 6-h measurement period. Participants brushed their teeth and rinsed their mouths with water 10 min prior to each sample collection to prevent sample contamination, as food was offered during study appointments. Participants were instructed to soak a cotton swab for 2–3 min, or until the swab was fully saturated (cotton swabs were weighed before and following saturation to ensure at least 2 ml of saliva were collected). Given the potential for the lab appointment to impact typical dietary patterns, dietary recalls were not collected for the day of or the days immediately preceding or following DLMO appointments.

### Measures

#### Covariates

##### Demographics

Information pertaining to potentially relevant covariates pertaining to sleep behaviors such as age, sex, race, and ethnicity were voluntarily collected via an online survey completed at the baseline appointment.

#### Predictors

##### Automated Self-Administered Dietary Assessment Tools (ASA24)

In order to measure dietary intake of the participants, the National Cancer Institute's Automated Self-Administered Dietary Assessment Tool was given on randomly assigned days during the study protocol. Participants were asked to enter their food, beverage, and supplement consumption for the preceding 24 h. The ASA24 offers estimates of the following micronutrients: calcium (calc), iron, magnesium (magn), potassium (pota), sodium (sodi), zinc, copper (copp), selenium (sele), vitamin C (VC), niacin (niac), vitamin B6 (VB6), folate (fola), folic acid (FA), retinol, vitamin K (VK), vitamin B1thiamin (VB1), vitamin B2 riboflavin (VB2), vitamin D (VitD), vitamin B12 (VB12), vitamin A, RAE, vitamin E (VE), alpha carotene (ACAR), beta carotene (BCAR), lycopene (lyco), cryptoxanthin beta (crypto), and lutein + zeaxanthin (LZ). Micronutrient intake data from this measure was used as predictors in statistical models. The ASA24 has been found to yield similar estimates to Automated Multiple-Pass Method (AMPM) standardized interviewer-administered recalls, with 87% of responses on the ASA24 falling within 20% variance of the AMPM (43). ASA24 is also comparable to in vivo food measurements, with participants in a buffet paradigm where food was weighed before and after consumption reporting 80% of items truly consumed on the ASA24, as compared with 83% in AMPM (44).

#### Outcomes

##### Sleep Behaviors

Sleep onset time, sleep midpoint time, total sleep duration, sleep efficiency, and WASO were derived from Actiwatch 2 data, with the understanding that adolescents are known to be unreliable reporters of their own sleep habits (45), and therefore behavioral measures of sleep may be valuable supplements to survey data. Previous studies have found that data from wrist-worn actigraphs strongly correlates with readings of polysomnography (*r* = 0.81–0.83), but specificity for wake times may be weaker (46, 47). To account for this potential weakness, actigraph readings were co-scored with participant sleep diaries, where time entering bed with intention to sleep, time initiating sleep, time of final awakening, time leaving the bed, and times waking during the night were self-reported. Actigraphy data was downloaded in 30 second epoch intervals and cleaned using ActiWatch software (Actiware 6.3). The onset and offset of each sleep period was cross-verified with sleep diaries. In the absence of a sleep diary, cases were double-scored by two reviewers, with bedtime defined as when the actigraph was donned (for those in the nighttime only monitoring sample) or as the beginning of the last downward trend of physical activity before sleep, and out-of-bedtime defined as the beginning of the first prolonged period of physical activity after prolonged inactivity associated with sleep (for those participating in the all-day monitoring protocol). The Sadeh algorithm (48) was used to calculate sleep onset, sleep offset, WASO, and sleep duration; sleep midpoint was then calculated as the halfway point between sleep offset and sleep onset. Sleep efficiency was calculated as: [sleep offset-sleep onset (time asleep)/time exiting bed-time entering bed (time in bed)]. All variables involving time were converted to minutes after midnight to prevent circular calculations.

##### Chronotype

The Morning-Eveningness Questionnaire (49) used to assess chronotype, or trait-like preference for early (morningness) or late (eveninngness) sleep and wake times. Higher scores reflect a morningness chronotype, while lower scores reflect an eveningness chronotype, with intermediate chronotypes falling in between. Although not specifically validated within adolescent populations, this 19-item self-report survey has been found to have good reliability in young adults (50) and adequate reliability within adolescents (51). Within our combined sample, the Cronbach's alpha ranged from 0.70–0.77, indicating adequate reliability.

##### Daytime Sleepiness

The Epworth Sleepiness Scale for Children and Adolescents (ESS-CHAD) was used to assess subjective sleepiness levels. This 36-item self-report measure asks adolescents to rate how likely they might be to doze off during common daily activities. Higher scores on this measure indicate higher levels of daytime sleepiness. The ESS-CHAD has been found to have strong test-retest reliability *(r* = 0.89) and internal validity (Chronbach's alpha = 0.73) in adolescents (52). Rasch analysis also indicates that the ESS-CHAD is interpretable as one dimension, with comparable model fit for the total sample, and for subgroups of males and females 7–9 and 10–12 (52), suggesting successful adaptation from the ESS for adults. Within our combined sample, the Cronbach's alpha was 0.863, indicating good internal reliability.

##### DLMO Time and Circadian Alignment

A total of 9–12 saliva samples were gathered across the 6-h DLMO procedure; samples were time-stamped, centrifuged at 3,000 g for 5 min to remove the saliva from the cotton swab, frozen, and later shipped to SolidPhase Inc. (an external lab) where they used Novolytix RIA kits to measure salivary concentrations for each sample. DLMO was determined as the time that saliva melatonin concentration levels exceeded 4 pg/mL. This threshold has been previously validated in adolescents (53). Circadian alignment, which is also known as phase angle, was derived from the difference in time between DLMO and average sleep onset time recorded via actigraphy across the monitoring period. All analyses were performed with continuous values. Clinically, these scores can be interpreted as “aligned” in cases of phase angle of > 2 and < 4 h, which can reflect the anticipated delay between DLMO and sleep initiation in low-light conditions, (54, 55) as well as “Misaligned” in cases where there is a time gap of < 2 h or > 4 h, which can indicate that adolescents are falling asleep significantly earlier or later than their body's natural, physiological readiness.

### Statistical analysis

All analyses were completed in R (56). No data points were excluded prior to analysis. As it is conceivable that some participants may not consume measurable levels of some micronutrients on any given day, missing datapoints for specific micronutrients (within otherwise completed recalls; *n* = 31 for day to night, *n* = 22 for average across days) were not excluded from analysis. Similarly, as it is conceivable that some adolescents will consume much more of any one micronutrient than their peers, yet represent typical eating patterns, no outliers in micronutrient consumption were removed before analysis.

To account for multiple dietary intakes per participant, we used the lme4 package (57) to complete a series of mixed effects models with random by-participant intercepts. The first series of models evaluated relationships between one day's dietary intake and the corresponding night's sleep; within these analyses, ASA-24 data was matched with the corresponding night's actigraphy. An additional series of linear regression models examined associations between average dietary intake across observed days and average sleep behavior within that monitoring period, as well as trait-like measures of sleep health such as chronotype and average daytime sleepiness. For these overall analyses, average intake was calculated by dividing total observed intake across all days by the number of dietary recalls collected for that participant. Similarly, average sleep behaviors were calculated by dividing total time for that sleep variable across all days by the number of days for which corresponding actigraphy for that participant was available.

All models included one sleep measurement as the outcome and, initially, all micronutrients as predictors. Due to recommended daily intake and limits varying across sexes, all analyses were performed separately for males and females. The dietary reference intakes for males and females differ due to distinct biological and metabolic needs (58). The *step* function from the lmerTest package (59) was used to perform backwards step-wise model fitting, removing micronutrients which did not improve the overall fit of the model. All final models showed Variance Inflation Factors less than five, indicating low collinearity, Fixed-effect estimates are interpreted as conditional associations within the observed dataset; inference was based on model-derived coefficient estimates and associated test statistics from the mixed-effects framework. *P*-values were corrected for multiple comparisons across all models (separately for daily and averaged models) using false discovery rate correction; Benjamini–Hochberg-adjusted *p-*values were calculated across all retained predictor–outcome tests.

## Results

### Participants

A total of 85 adolescents were recruited (57% self-reported assigned Female at birth, 87% non-Hispanic European; see Figure 1 and Table 1). All participants were between 14 and 18 years of age, with a mean age of 15.5 (SD = 1.24). Eighty participants completed at least one ASA-24 dietary recall and were included in final analyses. Fifty-three percent of the sample completed three dietary recalls and an additional 40% completed two dietary recalls. There were significant differences in all levels of micronutrient intake by sex for all micronutrients, save vitamin B12 and vitamin D (see Table 2). A greater proportion of females met daily recommendations on a variety of micronutrients compared to males, with the exception of vitamin C (see Table 2). Average levels of sodium, copper, selenium, vitamin C, niacin, vitamin B6, vitamin K, and vitamin B12 consumed by both males and females in our sample exceeded recommended daily intake (RDI) ranges for adolescents of both sexes (see Table 2). The average zinc, iron, and folate intake for males in our sample exceeded the recommended range for this group. All other mean micronutrient intake levels for our sample were below recommended ranges for each sex.

**Figure 1:**
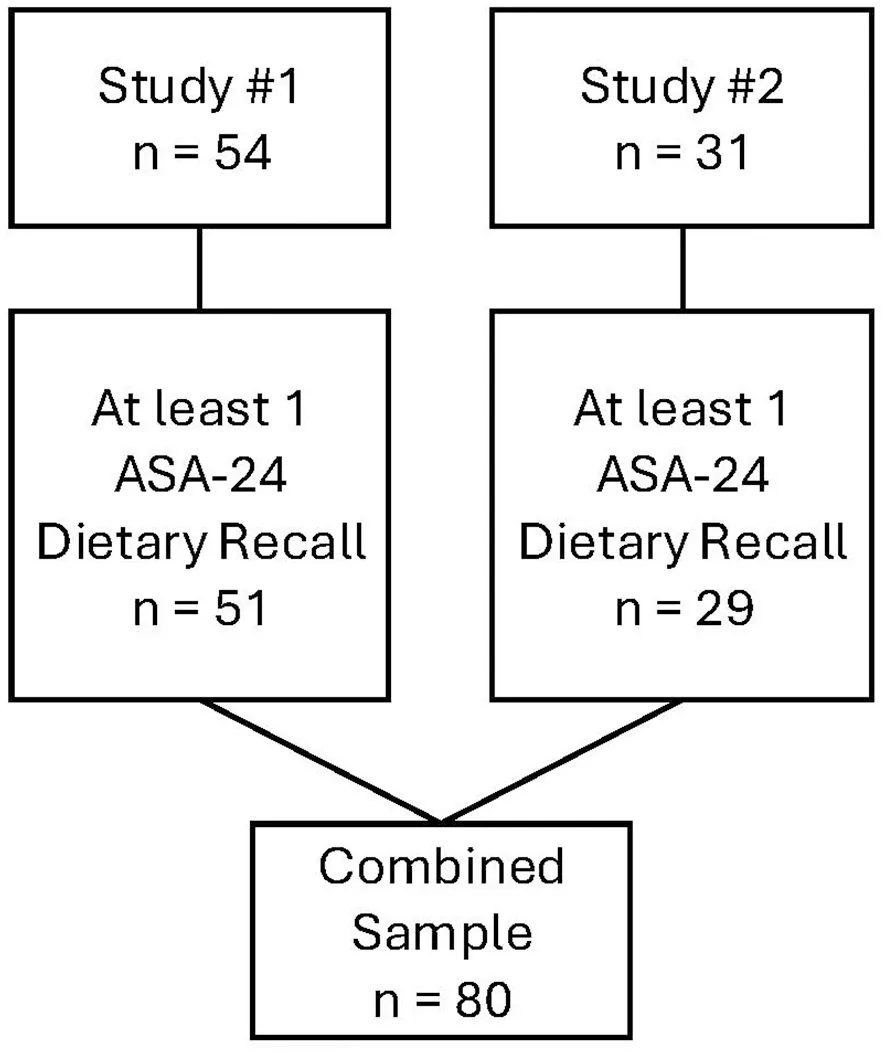
Overview of participants recruited and included in analysis.

**Table 1:** Participant sample characteristics.

Demographics	*N*	% Sample
*Race*	85	100%
Non-Hispanic European White	74	87.05%
Black	0	0.00%
Native American or Alaskan Native	3	3.53%
Asian or pacific Islander	6	7.06%
A race not listed	2	2.35%
*Ethnicity*	84	98.8%
Non-Hispanic, Latino or Spanish origin	74	88.10%
Hispanic, Latino or Spanish origin	10	11.90%
*Sex*	85	98.8%
Female	49	57.65%
Male	36	42.35%
Age^*^	*84*	
14	19	22.35%
15	26	30.95%
16	19	22.62%
17	11	13.10%
18	9	10.71%

^*^Mean age = 15.50 (SD = 1.24).

**Table 2:** Reported micronutrient intake by sex assigned at birth.

Micronutrient (unit)	FEMALE *N* = 49; Average Age: 15.59 years, 86% White, 8% Latinx				MALE *N* = 36; Average Age: 15.54 years, 89% White, 17% Latinx				*t* (p)
	**Average Reported Consumption (% of RDI)**	**Range Reported Consumption**	**RDI**	**% Sample Meeting RDI**	**Average Reported Consumption (% of RDI)**	**Range Reported Consumption**	**RDI**	**% Sample Meeting RDI**	**Difference in % RDI met by sex assigned at birth**
*CALC (mg)*	934.60 (72%)	79.78–3,411.71	1,300	39.51	1,279.5 (98%)	231.5–3,800.1	1,300	26.27	4.05 (0.000)^***^
*IRON (mg)*	13.16 (88%)	1.15–40.60	15	80.25	19.05 (173%)	4.88–54.97	11	28.81	7.58 (0.000)^***^
*MAGN (mg)*	241.9 (67%)	21.1–1,033.6	360	16.05	299.17 (73%)	78.25–606.1	410	16.10	2.07 (0.040)^*^
*POTA (mg)*	1,976.59 (42%)	99.92–5,538.44	4,700	7.41	2,665.6 (57%)	649.5–6,920.2	4,700	3.39	−2.72 (0.007)^**^
*SODI (mg)*	2,813.7 (122%)	193.7–7,460	2,300	80.25	3,708 (161%)	1,108–11,139	2,300	53.39	11.37 (0.000)^***^
*ZINC (mg)*	8.78 (98%)	0.5823–26.5814	9	61.73	14.991 (136%)	2.45–46.16	11	43.22	7.74 (0.000)^***^
*COPP (mg)*	0.98 (110%)	0.2381–3.4490	0.89	58.02	1.15 (129%)	0.41–3.22	0.89	54.24	9.55 (0.000)^***^
*SELE (mcg)*	90.2 (164%)	4.636–251.67	55	96.30	125.39 (228%)	18.97–412.31	55	78.81	14.94 (0.000)^***^
*VC (mg)*	78.09 (120%)	0.41–513.64	65	33.33	97.01 (129%)	0.29–1,191.77	75	46.61	4.50 (0.000)^***^
*NIAC (mg)*	17.38 (124%)	0.72–64.42	14	83.95	28.63 (179%)	8.07–62.12	16	57.63	11.63 (0.000)^***^
*VB6 (mg)*	1.47 (123%)	0.01–3.77	1.2	76.54	2.43 (187%)	0.36–7.2	1.3	51.69	10.27 (0.000)^***^
*FOLA (mcg)*	365.02 (91%)	22.21–1,233.97	400	54.32	491.65 (123%)	53.59–1,556.75	400	39.83	7.08 (0.000)^***^
*FA (mcg)*	192.5	0.0–784.2	–	–	287.5	0.0–1,390.2	–	–	–
*RET (mcg)*	430.7	0.0–1,800.6	–	–	684.85	11.17–2,397.38	–	–	–
*VK (mcg)*	87.94 (117%)	0.10–431.53	75	37.04	89.188 (119%)	1.43–528.57	75	41.53	6.44 (0.000)^***^
*VB1 (mg)*	1.42 (142%)	0.11–4.13	1.0	83.95	2.15 (179%)	0.26–7.64	1.2	72.03	12.34 (0.000)^***^
*VB2 (mg)*	1.74 (174%)	0.12–5.60	1.0	85.19	2.49 (192%)	0.5–5.94	1.3	75.42	15.44 (0.000)^***^
*VITD (mcg)*	7.39 (49%)	0.0–104.06	15	8.64	6.75 (45%)	0.00–28.48	15	8.47	−1.93 (0.055).
*VB12 (mcg)*	12.33 (514%)	0.07–1,002.79	2.4	85.19	7.07 (295%)	0.16–27.56	2.4	63.56	1.73 (0.085).
*RAE (μg)*	570.37 (81%)	10.25–1,928.56	700	35.80	824.6 (92%)	67.1–2,435.6	900	34.75	4.66 (0.000)^***^
*VE (mg)*	7.81	0.27–27.80	–	–	10.00	0.96–30.83	–	–	–
*ACAR (mcg)*	190.25	0.00–3,089.65	–	–	268.06	0.00–4,329	–	–	–
*BCAR (mcg)*	1,361.3	0.0–16,990	–	–	1,308.14	40.74–9,928.67	–	–	–
*LYCO (mcg)*	4,290	0–47,085	–	–	4,360	0.0–36,534	–	–	–
*CRYP (mcg)*	78.62	0.0–932.50	–	–	101.17	0.0–2,583.32	–	–	–
*LZ (mcg)*	1,196.82	3.69–9,825.96	–	–	1,201.7	104.9–11,895.3	–	–	–

0, “^***^”; 0.001, “^**^”; 0.01, “^*^”; 0.05, “.”. *p*-values after false discovery rate correction.

### Day's intake to night's sleep models

In the models examining relationships between daily dietary intake and the corresponding night's sleep behaviors, significant micronutrients were identified for each sex and sleep outcome. Full effects are outlined in Table 3 (male), Table 4 (female), and Figure 2 (all models).

**Table 3:** Day's micronutrient intake and same-night sleep domains for males.

Micronutrient (unit)	Efficiency (%) (*n* = 69)		Midpoint (min.) (*n* = 69)		Onset (min.) (*n* = 69)		WASO (min.) (*n* = 69)		Duration (min.) (*n* = 69)	
	Est.	SE	Est.	SE	Est.	SE	Est.	SE	Est.	SE
*CALC (mg)*	–	–	0.04^*^	0.02^*^	–	–	–	–	–	–
*IRON (mg)*	–	–	–	–	–	–	–	–	–	–
*MAGN (mg)*	–	–	–	–	–	–	–	–	–	–
*POTA (mg)*	0.002^*^	0.0007^*^	–	–	–	–	–	–	–	–
*SODI (mg)*	–	–	–	–	–	–	–	–	–	–
*ZINC (mg)*	–	–	–	–	3.51^**^	1.12^**^	–	–	–	–
*COPP (mg)*	–	–	–	–	–	–	−13.24^*^	5.96^*^	–	–
*SELE (mcg)*	−0.04^*^	0.02^*^	–	–	–	–	0.15^*^	0.06^*^	–	–
*VC (mg)*	–	–	–	–	–	–	–	–	–	–
*NIAC (mg)*	–	–	–	–	–	–	−1.53^***^	0.32^***^	–	–
*VB6 (mg)*	–	–	–	–	–	–	–	–	−14.11^*^	0.02^*^
*FOLA (mcg)*	–	–	–	–	–	–	−0.11^**^	0.03^**^	–	–
*FA (mcg)*	–	–	–	–	–	–	0.11^**^	0.03^**^	–	–
*RET (mcg)*	–	–	−0.20^***^	0.05^***^	−0.12^*^	0.04^*^	–	–	–	–
*VK (mcg)*	–	–	–	–	–	–	−0.18^**^	0.06^**^	–	–
*VB1 (mg)*	−1.10^*^	0.54^*^	–	–	–	–	12.08^***^	2.30^***^	–	–
*VB2 (mg)*	–	–	–	–	–	–	–	–	–	–
*VITD (mcg)*	–	–	–	–	–	–	−2.86^**^	0.74^**^	–	–
*VB12 (mcg)*	–	–	7.22^*^	2.60^*^	–	–	2.18^*^	0.85^*^	–	–
*RAE (μg)*	–	–	0.12^*^	0.04^*^	0.12^*^	0.04^*^	–	–	–	–
*VE (mg)*	–	–	–	–	–	–	1.50^**^	0.48^**^	–	–
*ACAR (mcg)*	–	–	–	–	–	–	–	–	–	–
*BCAR (mcg)*	–	–	–	–	–	–	–	–	–	–
*LYCO (mcg)*	–	–	–	–	–	–	0.002^***^	0.0005^***^	–	–
*CRYP (mcg)*	–	–	–	–	–	–	0.03^*^	0.01^*^	–	–
*LZ (mcg)*	–	–	–	–	–	–	0.01^*^	0.003^*^	–	–

0, “^***^”; 0.001, “^**^”; 0.01, “^*^”; 0.05, “.”. *p*-values after false discovery rate correction.

**Table 4:** Day's micronutrient intake and same-night sleep domains for females.

Micronutrient (unit)	Efficiency (%) (*n* = 99)		Midpoint (min.) (*n* = 99)		Onset (min.) (*n* = 99)		WASO (min.) (*n* = 99)		Duration (min.) (*n* = 99)	
	Est.	[SE]	Est.	[SE]	Est.	[SE]	Est.	[SE]	Est.	[SE]
*CALC (mg)*	–	–	–	–	–	–	0.02^*^	0.005^*^	–	–
*IRON (mg)*	–	–	–	–	–	–	–	–	–	–
*MAGN (mg)*	−0.04^*^	0.01^*^	–	–	–	–	–	–	–	–
*POTA (mg)*	0.004^*^	0.002^*^	–	–	−0.03^*^	0.01^*^	–	–	–	–
*SODI (mg)*	–	–	–	–	–	–	−0.004^*^	0.001^*^	−0.01^*^	0.005^*^
*ZINC (mg)*	–	–	–	–	–	–	–	–	–	–
*COPP (mg)*	–	–	–	–	–	–	–	–	–	–
*SELE (mcg)*	–	–	–	–	–	–	–	–	–	–
*VC (mg)*	−0.03^*^	0.01^*^	–	–	–	–	–	–	–	–
*NIAC (mg)*	–	–	–	–	–	–	–	–	–	–
*VB6 (mg)*	–	–	–	–	–	–	6.63^*^	2.79^*^	–	–
*FOLA (mcg)*	–	–	–	–	–	–	–	–	–	–
*FA (mcg)*	–	–	–	–	–	–	–	–	–	–
*RET (mcg)*	0.01^**^	0.003^**^	–	–	–	–	−0.02^*^	0.01^*^	–	–
*VK (mcg)*	–	–	0.63^**^	0.20^**^	0.75^**^	0.20^**^	–	–	–	–
*VB1 (mg)*	–	–	–	–	–	–	–	–	–	–
*VB2 (mg)*	–	–	–	–	–	–	–	–	–	–
*VITD (mcg)*	–	–	–	–	–	–	–	–	–	–
*VB12 (mcg)*	−0.96^*^	0.36^*^	–	–	–	–	–	–	–	–
*RAE (μg)*	–	–	–	–	0.06^*^	0.03^*^	–	–	–	–
*VE (mg)*	–	–	–	–	–	–	–	–	–	–
*ACAR (mcg)*	–	–	–	–	–	–	–	–	–	–
*BCAR (mcg)*	–	–	–	–	–	–	–	–	–	–
*LYCO (mcg)*	–	–	–	–	–	–	–	–	–	–
*CRYP (mcg)*	–	–	–	–	–	–	–	–	–	–
*LZ (mcg)*	0.002^*^	0.0007^*^	−0.04^**^	0.01^*^	−0.04^**^	0.01^**^	–	–	–	–

0, “^***^”; 0.001, “^**^”; 0.01, “^*^”; 0.05, “.” 0.1; *p*-values after false discovery rate correction.

**Figure 2:**
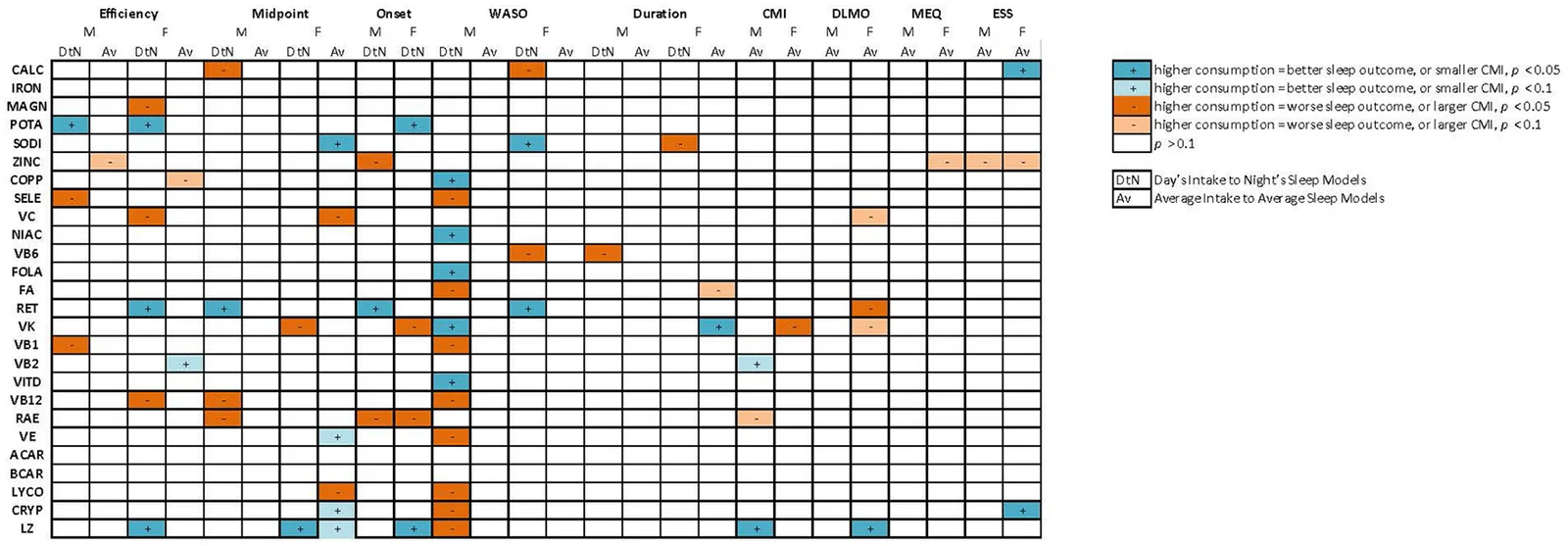
Summary of all findings by participant sex, model type, and sleep domain. Blue shading and + sign denotes improved sleep outcome with increased micronutrient intake; Orange shading and – sign denotes worsened sleep outcome with increased micronutrient intake.

#### Sleep efficiency

##### Males

Increased intake of potassium was associated with increased sleep efficiency that night in males (b = 0.002; *p* < 0.05), while increased intake of selenium and thiamin corresponded with decreased sleep efficiency in males (b's = −0.04 and−1.10; *p*'s < 0.05).

##### Females

Increased intake of retinol (b = 0.008, *p* < 0.01), Lutein + zeaxanthin (b = 0.002, *p* < 0.05), and potassium (b = 0.004, *p* < 0.05) was linked to increased sleep efficiency that night in females. Increased intake of magnesium (b = −0.04; *p* < 0.05), vitamin C (b = −0.03; *p* < 0.05*)* and vitamin B12 (b = −0.96; *p* < 0.05) was associated with decreased sleep efficiency in females.

#### Sleep midpoint

##### Males

Increased calcium (b = 0.04, *p* < 0.05), vitamin B12 (b = 7.22, *p* < 0.05), and vitamin A, RAE (b = 0.12, *p* < 0.05) consumption was associated with later sleep midpoint that night in males. Increased retinol consumption was associated with earlier sleep midpoint that night in males (b = −0.20, *p* < 0.001).

##### Females

Increased vitamin K consumption was associated with later sleep midpoint that night (b = 0.63, *p* < 0.01), while increased Lutein + zeaxanthin consumption was associated with earlier sleep midpoint (b = −0.04, *p* < 0.01) in females.

#### Sleep onset

##### Males

Increased zinc (b = 3.51, *p* < 0.01) and vitamin A, RAE (b = 0.12, *p* < 0.05) consumption was linked with later sleep onset times that night in males, while increased retinol was associated with earlier sleep onset times (b = −0.12, *p* < 0.05).

##### Females

As in males, increased vitamin A, RAE consumption was associated with later sleep onset times for that night in females (b = 0.06, *p* < 0.05). Increased vitamin K consumption also corresponded with later sleep onset time in females (b = 0.75, *p* < 0.01), while increased potassium (b = −0.03, *p* < 0.05) and Lutein + zeaxanthin consumption was linked with earlier sleep onset time (b = −0.04, *p* < 0.01).

#### Wake after sleep onset (WASO)

##### Males

Increased consumption of selenium (b = 0.15, *p* < 0.05), folic acid (b = 0.11, *p* < 0.01), thiamin (b = 12.08, *p* < 0.001, vitamin B12 (b = 2.18, *p* < 0.05), vitamin E (b = 1.50, *p* < 0.01), lycopene (b = 0.002, *p* < 0.001), cryptoxanthin beta (b = 0.03, *p* < 0.05), and Lutein + zeaxanthin (b = 0.01, *p* < 0.05) was linked with increased WASO that night in males. Increased consumption of copper (b = −13.24, *p* < 0.05), niacin (b = −1.53, *p* < 0.001), folate (b = −0.11, *p* < 0.01), vitamin K (b = −0.18, *p* < 0.01), and vitamin D (b = −2.86, *p* < 0.01) was associated with decreased WASO that night.

##### Females

Increased consumption of calcium (b = 0.02, *p* < 0.05) and vitamin B6 (b = 6.63, *p* < 0.05) was associated with increased WASO that night in females, while increased consumption of sodium (b = −0.004, *p* < 0.05) and retinol (b = −0.02, *p* < 0.05) was linked with decreased WASO that night.

#### Sleep duration

##### Males

Increased consumption of vitamin B6 was linked with decreased sleep duration that night in males (b = −14.11, *p* < 0.05).

##### Females

Increased levels of sodium consumption was associated with decreased sleep duration the same night in females (b = −0.01, *p* < 0.05).

### Average intake to average sleep models

In the models examining relationships between average dietary intake across all observed days and average sleep behaviors across all observed nights, as well as on more trait-like measures of sleep health such as average daytime sleepiness, chronotype, DLMO time, and circadian misalignment status, significant micronutrients were identified for each sex and sleep outcome. Full effects are outlined in Table 5 (male), Table 6 (female), and Figure 2 (all models).

**Table 5:** Average micronutrient intake and average and baseline sleep domains for males.

Micronutrient (unit)	Efficiency (*n* = 31)		Midpoint (*n* = 31)		CMI (*n* = 31)		WASO (*n* = 31)		Duration (*n* = 31)		DLMO (*n* = 31)		MEQ (*n* = 33)		ESS (*n* = 31)	
	Est.	[SE]	Est.	[SE]	Est.	[SE]	Est.	[SE]	Est.	[SE]	Est.	[SE]	Est.	[SE]	Est.	[SE]
*CALC (mg)*	–	–	–	–	–	–	–	–	–	–	–	–	–	–	–	–
*IRON (mg)*	–	–	–	–	–	–	–	–	–	–	–	–	–	–	–	–
*MAGN (mg)*	–	–	–	–	–	–	–	–	–	–	–	–	–	–	–	–
*POTA (mg)*	–	–	–	–	–	–	–	–	–	–	–	–	–	–	–	–
*SODI (mg)*	–	–	–	–	–	–	–	–	–	–	–	–	–	–	–	–
*ZINC (mg)*	−0.32^.^	0.13^.^	–	–	–	–	–	–	–	–	–	–	–	–	0.32^.^	0.14^.^
*COPP (mg)*	–	–	–	–	–	–	–	–	–	–	–	–	–	–	–	–
*SELE (mcg)*	–	–	–	–	–	–	–	–	–	–	–	–	–	–	–	–
*VC (mg)*	–	–	–	–	–	–	–	–	–	–	–	–	–	–	–	–
*NIAC (mg)*	–	–	–	–	–	–	–	–	–	–	–	–	–	–	–	–
*VB6 (mg)*	–	–	–	–	–	–	–	–	–	–	–	–	–	–	–	–
*FOLA (mcg)*	–	–	–	–	–	–	–	–	–	–	–	–	–	–	–	–
*FA (mcg)*	–	–	–	–	–	–	–	–	–	–	–	–	–	–	–	–
*RET (mcg)*	–	–	–	–	–	–	–	–	–	–	–	–	–	–	–	–
*VK (mcg)*	–	–	–	–	–	–	–	–	–	–	–	–	–	–	–	–
*VB1 (mg)*	–	–	–	–	–	–	–	–	–	–	–	–	–	–	–	–
*VB2 (mg)*	–	–	–	–	55.08^.^	26.34^.^	–	–	–	–	–	–	–	–	–	–
*VITD (mcg)*	–	–	–	–	–	–	–	–	–	–	–	–	–	–	–	–
*VB12 (mcg)*	–	–	–	–	–	–	–	–	–	–	–	–	–	–	–	–
*RAE (μg)*	–	–	–	–	−0.15^.^	0.06^.^	–	–	–	–	–	–	–	–	–	–
*VE (mg)*	–	–	–	–	–	–	–	–	–	–	–	–	–	–	–	–
*ACAR (mcg)*	–	–	–	–	–	–	–	–	–	–	–	–	–	–	–	–
*BCAR (mcg)*	–	–	–	–	–	–	–	–	–	–	–	–	–	–	–	–
*LYCO (mcg)*	–	–	–	–	–	–	–	–	–	–	–	–	–	–	–	–
*CRYP (mcg)*	–	–	–	–	–	–		–	–	–	–	–	–	–	–	–
*LZ (mcg)*	–	–	–	–	0.02^*^	0.007^*^	–	–	–	–	–	–	–	–	–	–

0, “^***^”; 0.001, “^**^”; 0.01, “^*^”; 0.05, “.”. *p*-values after false discovery rate correction.

**Table 6:** Average micronutrient intake and average and baseline sleep domains for females.

Micronutrient (unit)	Efficiency (*n* = 39)		Midpoint (*n* = 39)		CMI (*n* = 33)		WASO (*n* = 39)		Duration (*n* = 39)		DLMO (*n* = 38)		MEQ (*n* = 45)		ESS (*n* = 44)	
	Est.	[SE]	Est.	[SE]	Est.	[SE]	Est.	[SE]	Est.	[SE]	Est.	[SE]	Est.	[SE]	Est.	[SE]
*CALC (mg)*	–	–	–	–	–	–	–	–	–	–	–	–	–	–	−0.00^*^	0.00^*^
*IRON (mg)*	–	–	–	–	–	–	–	–	–	–	–	–	–	–	–	–
*MAGN (mg)*	–	–	–	–	–	–	–	–	–	–	–	–	–	–	–	–
*POTA (mg)*	–	–	–	–	–	–	–	–	–	–	–	–	–	–	–	–
*SODI (mg)*	–	–	−0.04^*^	0.01^*^	–	–	–	–	–	–	–	–	–	–	–	–
*ZINC (mg)*	–	–	–	–	–	–	–	–	–	–	–	–	−0.54.	0.26.	0.72^.^	0.32.
*COPP (mg)*	−4.89^.^	2.12^.^	–	–	–	–	–	–	–	–	–	–	–	–	–	–
*SELE (mcg)*	–	–	–	–	–	–			–	–	–	–	–	–		
*VC (mg)*	–	–	1.46^*^	0.39^*^	–	–	–	–			0.37^.^	0.15^.^	–	–	–	–
*NIAC (mg)*	–	–	–	–	–	–	–	–	–	–	–	–	–	–	–	–
*VB6 (mg)*	–	–	–	–	–	–	–	–	–	–	–	–	–	–	–	–
*FOLA (mcg)*	–	–	–	–	–	–	–	–	–	–	–	–	–	–	–	–
*FA (mcg)*	–	–	–	–	–	–	–	–	−0.17^.^	0.07^.^	–	–	–	–	–	–
*RET (mcg)*	–	–	–	–	–	–	–	–	–	–	–	–	–	–	–	–
*VK (mcg)*	–	–	–	–	−1.07^**^	0.22^**^	–	–	0.67^*^	0.20^*^	1.28^.^	0.55^.^	–	–	–	–
*VB1 (mg)*	–	–	–	–	–	–	–	–	–	–	–	–	–	–	–	–
*VB2 (mg)*	3.1^.^	1.35^.^	–	–	–	–	–	–	–	–	–	–	–	–	–	–
*VITD (mcg)*	–	–	–	–	–	–	–	–	–	–	–	–	–	–	–	–
*VB12 (mcg)*	–	–	–	–	–	–	–	–	–	–						
*RAE (μg)*	–	–	–	–	–	–	–	–	–	–	–	–	–	–	–	–
*VE (mg)*	–	–	−10.29^.^	3.93^.^	–	–	–	–	–	–	–	–	–	–	–	–
*ACAR (mcg)*	–	–	–	–	–	–	–	–	–	–	–	–	–	–	–	–
*BCAR (mcg)*	–	–	–	–	–	–	–	–	–	–	–	–	–	–	–	–
*LYCO (mcg)*	–	–	0.00^*^	0.00^*^	–	–	–	–	–	–	–	–	–	–	–	–
*CRYP (mcg)*	–	–	−0.35^*^	0.17^*^	–	–	–	–	–	–	–	–	–	–	−0.03^*^	0.00^*^
*LZ (mcg)*	–	–	−0.04^*^	0.02^*^	–	–	–	–	–	–	−0.07^*^	0.02^*^	–	–	–	–

0, “^***^”; 0.001, “^**^”; 0.01, “^*^”; 0.05, “.”. *p*-values after false discovery rate correction.

#### Average sleep efficiency

##### Males

Increased averaged zinc consumption trended toward significant association with decreased averaged sleep efficiency in males (b = −0.32, *p* < 0.1).

##### Females

Increased average copper consumption in females trended toward a significant relationship with decreased sleep efficiency (b = −4.89, *p* < 0.1), while increased riboflavin (b = 3.1, *p* < 0.1) consumption trended toward a significant relationship with increased sleep efficiency.

#### Average sleep midpoint

##### Males

There was no association between intake and averaged sleep midpoint in males.

##### Females

Increased averaged vitamin C (b = 1.46, *p* < 0.05), and lycopene (b = 0.008, *p* < 0.05) consumption was significantly linked with later average sleep midpoint for females, while increased averaged sodium (b = −0.04, *p* < 0.05), vitamin E (b = −10.29, *p* < 0.1), cryptoxanthin beta (b = −0.35, *p* < 0.1), and lutein + zeaxanthin (b = −0.04, *p* < 0.5) consumption intake neared or reached significance for association with earlier average sleep midpoint. Of note, findings were similar for single-day and overall average models of lutein + zeaxanthin and sleep midpoint within this group.

#### Circadian misalignment index

##### Males

Increased averaged intake of riboflavin (b = 55.08, *p* < 0.1) and lutein + zeaxanthin (b = −0.02, *p* < 0.05) was significantly associated with or trended toward higher phase angle (greater time between DLMO and sleep onset time in males), while increased averaged intake of vitamin A, RAE (b = −0.15, *p* < 0.1) trended toward smaller phase angle (shorter time between DLMO and sleep onset).

##### Females

Increased levels of overall vitamin K consumption were linked with smaller phase angle (less time between DLMO and sleep onset; b = −1.07, *p* < 0.01).

#### Average wake after sleep onset (WASO)

No significant associations were identified between averaged micronutrient intake and average WASO in males or females.

#### Average sleep duration

##### Males

No significant associations were identified between overall micronutrient intake and average sleep duration in males.

##### Females

Increased average vitamin K consumption was significantly associated with increased average sleep duration in females (b = 0.67, *p* < 0.05). Increased folic acid consumption trended toward an association with decreased average sleep duration for females (b = −0.17, *p* < 0.1).

#### Dim light melatonin onset (DLMO) time

##### Males

No significant associations were identified between overall micronutrient intake and DLMO time in males.

##### Females

Increased average consumption of vitamin C (b = 0.37, *p* < 0.1) and vitamin K (b = 1.28, *p* < 0.1) trended toward association with later DLMO time in females. Conversely, increased average consumption of lutein + zeaxanthin (b = −0.07, *p* < 0.05) was significantly linked with earlier DLMO time in females.

#### Chronotype

##### Males

No significant associations were identified between overall micronutrient intake and DLMO time in males.

##### Females

Increased consumption of zinc trended toward association with lower MEQ scores (greater tendency toward eveningness) in females (b = −0.54, *p* < 0.1).

#### Daytime sleepiness

##### Males

Increased average consumption of zinc trended toward association with increased levels of daytime sleepiness in males (b = 0.32, *p* < 0.1).

##### Females

As in males, increased average consumption of zinc trended toward relation to increased levels of daytime sleepiness in females (b = 0.72, *p* < 0.1). On the other hand, increased average consumption of calcium (b = −0.006, *p* < 0.05) and cryptoxanthin beta (b = −0.03, *p* < 0.05) was associated with decreased levels of daytime sleepiness in females.

## Discussion

This study evaluated how adolescents' daytime intake of micronutrients was related to same-night sleep outcomes. We also explored how average micronutrient intake across the measurement period was associated with averaged (multi-day) or more trait-like (single timepoint measurement of trait-like domains) sleep health outcomes. In contrast with our initial hypotheses, no micronutrient emerged as significantly associated with improved sleep health across all models. Potassium and retinol were most consistently linked with positive sleep health outcomes across multiple models. Specifically, higher daily potassium intake was linked with increased same-night sleep efficiency for males and females alike. It was also associated with earlier same-night sleep onset for females. These findings are in line with those of a classic double-blind, placebo-controlled, crossover study in nine adult males on a potassium-deficient diet, where potassium supplementation was linked with improved sleep efficiency and sleep consolidation (34). More recent studies have linked lower potassium intake (indicated by lower urinary potassium excretion) to increased levels of daytime sleepiness in adults (60) and lower same-night sleep quality in adult women (75). More behaviorally, lower dietary intake of potassium has been associated with higher levels of insomnia (61). Interestingly, Okamoto et al.'s (61) analyses of food intake logs identified potassium intake at dinnertime as the most crucial window for improving insomnia symptoms. This is in line with this analysis with our findings showing the most robust associations between potassium and sleep in the day-to-night models as opposed to averaged over time models. This can suggest that sleep may be sensitive to day-to-day shifts in potassium intake, in addition to theorized effects of chronically inadequate intake, though future research is needed.

Although retinol (a specific chemical form of vitamin A) was consistently linked with improved sleep across several models, there was not clear replication of findings between sexes or sleep domains. Notably, all findings were observed in the day-to-night models, as opposed to average intake models. The positive associations between retinol and efficiency (females), midpoint (males), onset (males), and WASO (females) suggest a potentially broad, though context-dependent, role of retinol in supporting sleep health. A study of dermatology patients ages 16–40 years also found that taking isotretinoin, which is a derivative of vitamin A (as is retinol), was linked to improved sleep efficiency and decreased sleep latency after one month (62). A recent review paper highlights how these findings have been expanded upon over the last two decades, within a limited, but growing literature base (63). However, daily RAE (a standardized measure of total vitamin A activity from both retinol and carotenoid sources) was associated with *later* midpoint (males) and *later* sleep onset (males and females). This suggests potential nuance in relationships between different forms of vitamin A and sleep health.

Vitamin D was also associated with improved sleep in one of our models, namely, for reducing same-night WASO in males. Within children and adolescents, vitamin D deficiency has been consistently associated with both decreased sleep duration and poorer sleep efficiency (64). While overall vitamin D status was not directly measured within this study, over half of our participants did not meet recommended dietary intake for vitamin D. Other significant sleep associations may have emerged for longer term patterns of intake and deficiency.

Two micronutrients demonstrated variable positive or negative relationships with many sleep domains. Lutein + Xanthin was associated with both positive and negative sleep outcomes, with negative associations observed only in male models, and positive associations observed in female models. Consumption in females was associated with an earlier sleep midpoint on both the same night as well as averaged midpoint across the entire week; previously, Lutein + Zeaxanthin has been shown to promote optimal sleep in adults (65). Findings for vitamin K were also mixed, with only one positive association observed for males, and multiple positive and negative associations observed for females. In terms of positive effects, increased daily vitamin K consumption in females was associated with decreased wakefulness that night; greater vitamin K consumption was also associated with longer sleep duration across the averaged week. These aligns with previous findings in adults where vitamin K deficiency was associated with decreased sleep duration (21). However, some research suggests that the positive effects of vitamin K diminish after surpassing a minimal intake threshold (66). In our sample, both male and female mean consumption surpassed the RDA, which may account for variability in observed findings, as more clearly protective effects could be observed in the context of suboptimal intake.

Several micronutrients also demonstrated consistently negative associations with sleep health. Zinc emerged as the micronutrient most consistently linked with negative sleep health, with five separate models demonstrating increased consumption relating to poorer sleep. Directionality of these effects was consistent across both single-day (male day-to-night sleep onset, male and female average daytime sleepiness) and multi-day sleep health domains (male average sleep efficiency, female chronotype). A recent systemic review observed that responses to zinc supplementation did seem to vary by age group, with zinc positively impacting sleep quality in infants, and yielding no effect on sleep quality in some adult samples (67); however, supplementation of zinc is different than obtaining zinc from food sources, suggesting that future research on evaluating the relationship between zinc (derived from its various sources) and sleep across development is warranted.

Greater intake of vitamin B12 was also associated with poorer sleep across three models. In our sample, mean intake of vitamin B12 surpassed the RDA three-fold for males, and five-fold for females. This pattern of higher vitamin B12 intake corresponding with worse sleep health is consistent with multiple studies around vitamin B12 having an “alerting” effect, increasing likelihood later sleep onset and reduced sleep duration (68). Although using different methodology than our dietary intake reports, other studies suggest that lower serum levels of vitamin B12 correspond with greater insomnia symptoms (23, 68). Similarly, increased vitamin C was associated with poorer sleep across three separate models, with the mean intake being ~120% greater than the RDA. Contrary to our findings, a review including 17 papers on vitamin C and sleep found converging evidence for vitamin C alleviating some insomnia symptoms and sleep apnea symptoms in adults (69). However, many participants in these studies were clinical, hospital populations, which are likely to differ from our sample of general population adolescents.

Based on the adult literature (17–22) we also expected to observe robust protective effects for iron (though none was observed), magnesium (lower day-to-night sleep efficiency in female), calcium (lower daytime sleepiness in females, later day-to-night sleep midpoint in males, more same-night wake after sleep onset in females), and folic acid (increased same-night wake after sleep onset and overall circadian misalignment for males). Given that our female sample was on average below RDA for iron, magnesium, and calcium, and our male sample was below threshold for calcium and magnesium, it is possible that we were not able to detect effects of adequate or excess consumption.

Of note, many factors influence the absorption and metabolism of vitamins and minerals and must be considered when interpreting the data. The absorption of carotenoids (β-carotene, β-cryptoxanthin, lycopene, lutein, and zeaxanthin) is less than vitamin A (retinol, retinal, retinoic acid). People with low storage of some vitamins and minerals such as vitamin A, calcium, and iron increase their ability to absorb more of these nutrients. The nutrients also interact with one another either inhibiting or enhancing absorption, meaning that individual adolescents in our sample may have exhibited different patterns of absorption, related to individual patterns of food and supplement consumption. An individualized approach to treatment of understanding the source of micronutrient intake (*in vivo* consumption vs. supplementation) and specific micronutrient deficiencies is vital in all contexts, including within context of sleep health, even in domains where replicating patterns were observed. Further, it is possible that particular combinations of micronutrient supplementation, rather than supplementation of any micronutrient in isolation, may be most beneficial. Future studies can examine relationships between micronutrients, and evaluate more specific relationships between food and supplement sources of micronutrients, in addition to considering them as separate variables, as we have done in this first-pass, exploratory analysis.

There are also many nuances in interactions between sleep health domains. In general, our findings appeared to be most robust for aspects of sleep health related to daily behaviors, as opposed to more intrinsic traits. They also seemed to correspond most to adequate sleep duration, as opposed to timing of sleep. However, sleep domains are inherently interrelated; for example, changes in sleep efficiency or WASO can directly impact overall duration, just as shifts in sleep timing may influence the alignment between internal circadian rhythms and environmental demands. As such, some of the variability in our findings may reflect cascading effects across multiple sleep dimensions. Future studies would benefit from modeling these domains simultaneously or examining potential mediating and moderating pathways between micronutrient intake and broader patterns of sleep health.

While this study provides valuable knowledge and insight on adolescent micronutrient intake and adolescent sleep health, several limitations must be acknowledged. The study's cross-sectional design limits the ability to assess causal relationships, though our use of daily intake to same night sleep helps establish temporal precedence in some analyses. Additionally, the use of self-reported dietary intake measures introduces potential biases (desirability bias, recall bias) and self-report errors, which may affect data accuracy. Further, a number of relationships were compared across all models, which increases the risk for Type 1 error. We addressed this with a false discovery rate correction. As this is the first analysis of this kind in this age group, we also reported on models trending toward statistical significance (*p* < 0.1) in addition to those reaching statistical significance (*p* < 0.05), as findings of null effect also require replication before any conclusions about the relationship between any micronutrient and sleep health in this cohort can be drawn. Further, many relationships reported in previous adult studies fell between corrected and uncorrected significance values within our sample. Given the secondary nature of all our models, and the lack of established effect sizes which would inform a priori power analyses, all findings we report should be interpreted as exploratory signals of potential association requiring confirmation in independently powered studies.

In terms of generalizability of our findings, it is also important to note that our sample was predominately Non-Hispanic European White. Future studies should continue to recruit more representative samples, especially as dietary intake is known to vary across cultures (70). Larger samples could also improve ability to detect changes in effects across total caloric intake ranges, weekends vs. weekdays, school- versus summer periods, income levels, and/or body mass indices.

Despite its limitations, this study remains an important exploration of dietary patterns and sleep during adolescence, which is a critical period for habit formation, development, and susceptibility to risky health behaviors. Furthermore, this study has several substantial strengths. ASA-24 dietary recalls exhibit strong compatibility with the Automated Multiple-Pass Method (AMPM) interviewer-administered recalls (43, 44), demonstrating accuracy in capturing adolescents' dietary intake. In addition, the comprehensive inclusion of 26 micronutrients in the ASA-24 recalls and our analysis allows for a thorough exploration of carotenoids, minerals, and vitamins. The study design included the collection of 2–3 dietary recalls per participant, capturing both weekday and weekend variability to provide a more representative sample of typical dietary patterns for robust analysis (as compared with existing research with only one day of dietary recall). In addition to the dietary measures, the use of multimodal sleep assessment provides a holistic view of adolescent sleep patterns in relation to micronutrient intake.

## Conclusions

A key intention of sharing exploratory analyses, even with limitations, is to encourage other labs to use dietary recall data they may already have collected or plan to collect to continue examining relationships between micronutrients and sleep, which are understudied in youth. Our findings suggest that micronutrient intake is related to multiple, distinct domains adolescent sleep health. Results support short-term, day-to-night associations between dietary intake and same-night sleep, while also pointing to potentially different relationships between multi-day intake patterns and multi-day and trait-like sleep outcomes. Our adolescent-focused sample also revealed some notable deviations from the existing adult literature, highlighting the importance of further research across developmental periods. Further studies are warranted to better understand the impacts of chronic under- and over-consumption, as well as to identify optimal intake ranges for sleep health. Experimental and longitudinal designs will be critical for clarifying causal pathways, which can contribute to development of targeted, evidence-based interventions. Our findings do not identify any universal, one-size-fits-all solution for improving adolescent sleep through dietary intake alone. Interventions studied in the future will likely need to be tailored, taking into account individual intake patterns and associated immediate or storage-level deficiencies, timing of intake, and interactions between multiple nutrients to fully capture micronutrients' potential to promote sleep via known and theorized antioxidant, parasympathetic, neurotransmission, or other pathways (27, 71–74).

## Data Availability

The raw data supporting the conclusions of this article will be made available by the authors, without undue reservation.
